# A case of primary intestinal lymphangiectasia with non-Hodgkin lymphoma

**DOI:** 10.1186/s12876-021-01997-x

**Published:** 2021-12-11

**Authors:** Doudou Hu, Xianghua Cui, Wanlei Ren, Jian Zhang, Xin Guan, Xiangjun Jiang

**Affiliations:** 1 Department of Gastroenterology, Qingdao Municipal Hospital, Jiaozhou Road 1#, Qingdao, 266071 People’s Republic of China; 2 Department of Traditional Chinese Medicine, Qingdao Central Hospital, Qingdao, 266042 People’s Republic of China

**Keywords:** Primary intestinal lymphangiectasia, Lymphoma, Protein-losing enteropathy

## Abstract

**Background:**

Primary intestinal lymphangiectasia (PIL) is a rare protein-losing enteropathy characterized by the loss of proteins, lymphocytes, and immunoglobulins into the intestinal lumen. Increasing evidence has demonstrated an association between PIL and lymphoma.

**Case presentation:**

A 54-year-old man with a 20-year history of abdominal distension and bilateral lower limb edema was admitted. Laboratory investigations revealed lymphopenia, hypoalbuminemia, decreased triglyceride and cholesterol level. Colonoscopy showed multiple smooth pseudo polyps in the ileocecal valve and terminal ileum and histological examination showed conspicuous dilation of the lymphatic channels in the mucosa and submucosa. A diagnosis of PIL was made. Three years later colonoscopy of the patient showed an intraluminal proliferative mass in the ascending colon and biopsy examination confirmed a malignant non-Hodgkin lymphoma. Then the patient was been underwent chemotherapy, and his clinical condition is satisfactory.

**Conclusion:**

Our report supports the hypothesis that PIL is associated with lymphoma development.

## Background

Primary intestinal lymphangiectasia (PIL) is a rare disease manifested by the loss of proteins, lymphocytes, and immunoglobulins into the intestinal lumen [1], which in turn leads to the suppression of both the humoral and cellular immune systems [2]. Since the first description in 1961 [3], nearly 200 cases of PIL cases have been reported globally [4]. However, with the improvement of endoscopic technology, especially the enteroscopy, PIL is increasingly been recognized and the prevalence might be underestimated. And increasing evidence has demonstrated an association between PIL and lymphoma [2, 5–10]. Here, we report a case of diffuse large B-cell lymphoma occurring 23 years after the onset of PIL, and conduct a systematic review of the literature on lymphoma and PIL.

## Case presentation

**Fig. 1:**
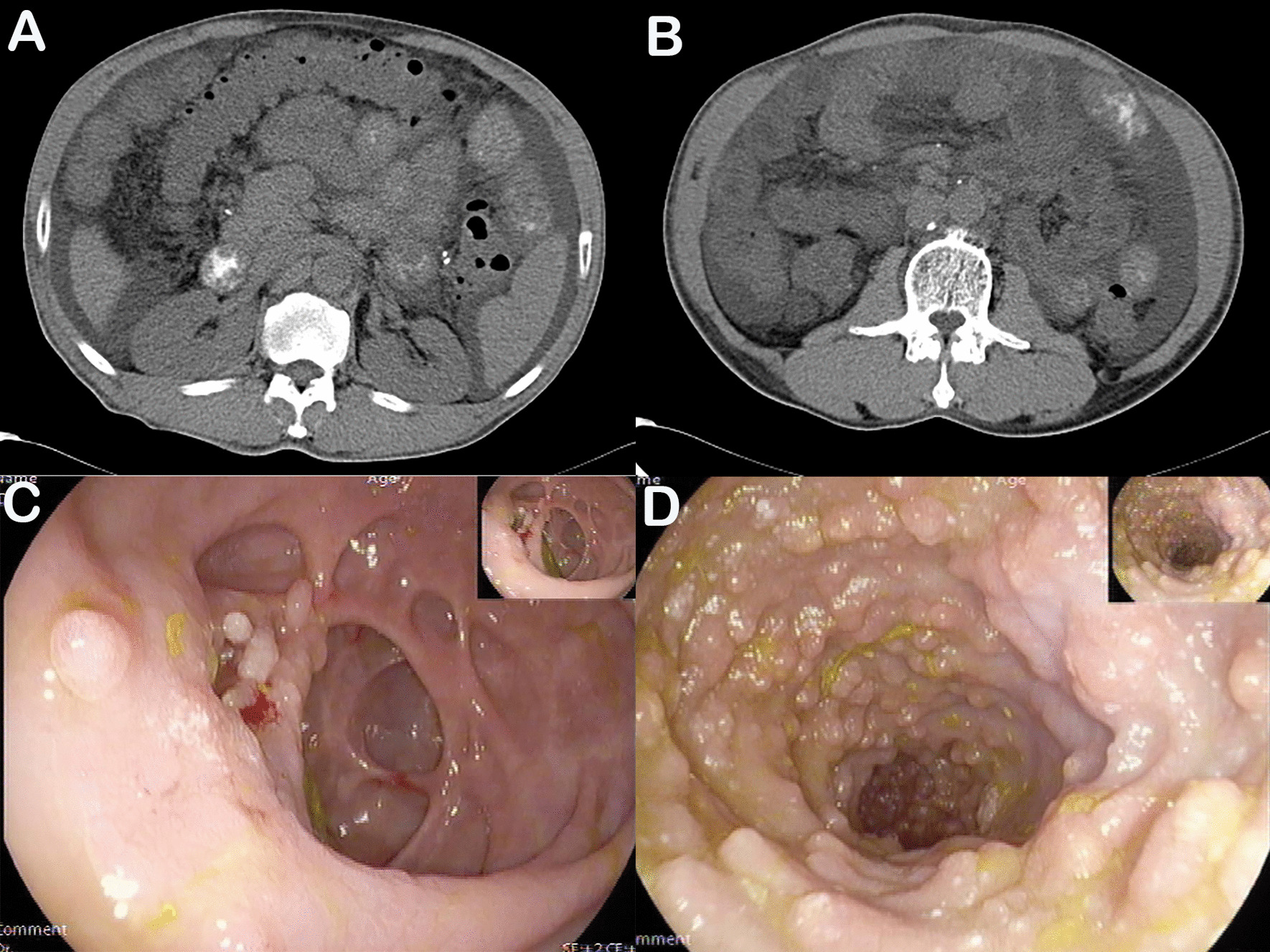
Plain computed tomography shows ascites and diffuse thickening of multiple small bowel loops (**a, b**). Endoscopic views of the ileocecal valve (**c**) and terminal ileum (**d**)

**Fig. 2:**
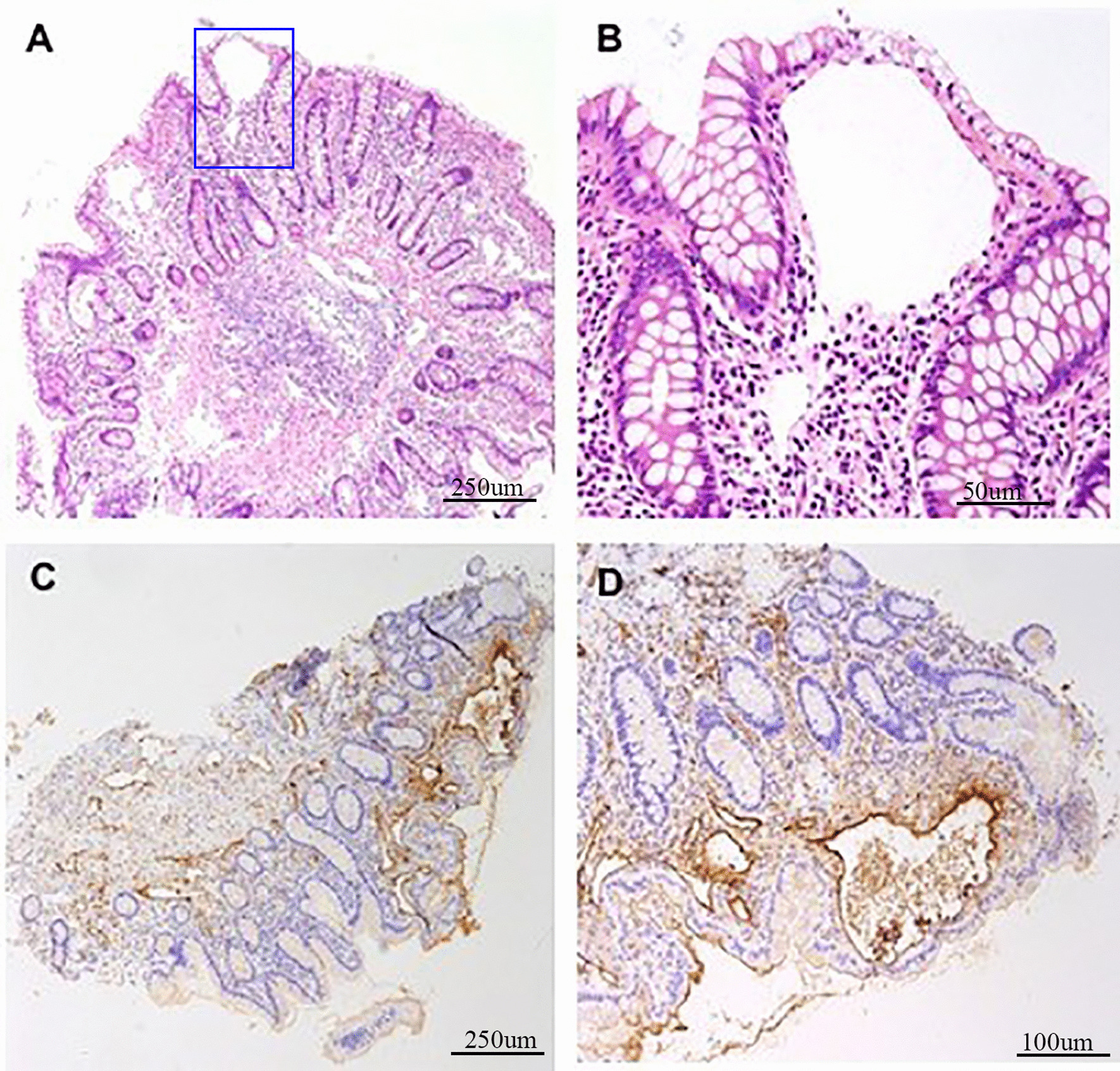
Biopsy examination reveals marked submucosal lymphangiectasia. **a, b** Hematoxylin and eosin staining. Magnification, 40-fold magnification (**a**) and 200-fold magnification (**b**). **c, d** D-240 staining. Magnification, 40-fold magnification (**c**) and 100-fold magnification (**d**)

In January 2016, a 54-year-old man with a 20-year history of abdominal distension and bilateral lower limb edema was admitted to our hospital. The patient reported no history of coronary atherosclerotic heart disease, kidney disease, thyroid disease, or hepatitis. A physical examination confirmed the presence of abdominal distension and pitting edema in both lower limbs. Laboratory investigations revealed the following: lymphopenia (lymphocyte count, 0.45*10^9^/L; normal range 1.1–3.2*10^9^/L), hypoalbuminemia (albumin, 15.8 g/L; normal range 40–55 g/L), decreased triglyceride level (0.36 mmol/L; normal range 0.4–1.8 mmol/L), decreased cholesterol level (2.47 mmol/L; normal range 3.6–6.5 mmol/L), and normal liver and kidney function. Abdominal computed tomography revealed a small amount of fluid and diffuse thickening of multiple small bowel loops (Fig. 1a, b). The patient then underwent colonoscopy, which revealed multiple smooth pseudo polyps in the ileocecal valve and terminal ileum (Fig. 1c, d). Biopsies were taken from ileocecal valve and terminal ileum to exclude lymphoma and intestinal lymphangiectasia. The lymphatic channels in the mucosa and submucosa were dilated conspicuously and D2-40 positive staining, a marker of lymphatic endothelial cells, was observed (Fig. 2). The diagnosis of primary intestinal lymphangiectasia (PIL) was made, and the patient was immediately prescribed a low-fat, high-protein diet and medium-chain triglyceride supplementation.

**Fig. 3:**
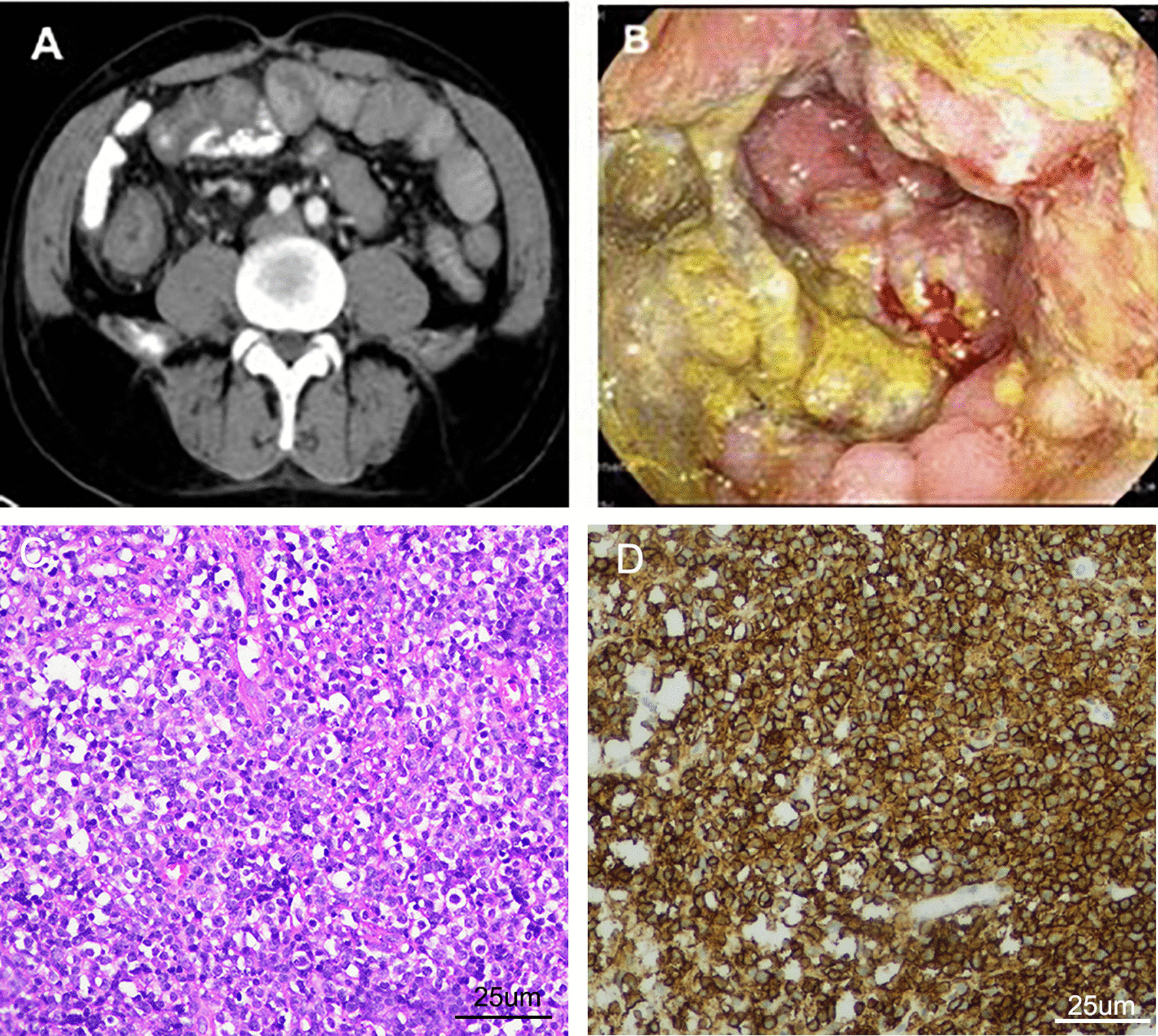
**a** Contrast-enhanced computed tomography and **b** colonoscopy show a mass lesion in the ascending colon. **c** A biopsy examination reveals a non-Hodgkin malignant lymphoma (hematoxylin and eosin staining; magnification, 400-fold magnification). **d** A biopsy examination reveals a non-Hodgkin malignant lymphoma (CD20 staining; magnification, 400-fold magnification)

On May 5, 2019, the patient returned to our hospital with a right-sided inguinal hernia that had been present for 6 months. A physical examination showed a 3*3-cm mass in the right inguinal region. Abdominal computed tomography showed thickening of the ascending colon with luminal narrowing (Fig. 3a). Colonoscopy showed an intraluminal proliferative mass in the ascending colon (Fig. 3b). Biopsy examination confirmed a malignant non-Hodgkin lymphoma (Fig. 3c, d). The tumor cells stained positive for CD20 (diffuse, strong), CD79a (diffuse+), Ki67 (90%), BCL6 (+), CD10 (weak, sun), BCL6 (+), and c-Myc (30%), and negative for CD5, cyclinD1, CD23, CD21, BCL2, MUM1, CD30, and p53 (wild type), which was consistent with a diagnosis of diffuse large B-cell lymphoma. In situ hybridization test showed that the tumor cells were negative for the Epstein-Barr encoding region (EBER).

Chemotherapy with the R-CHOP regimen (rituximab, cyclophosphamide, doxorubicin, vincristine, and prednisone) was initiated. At the time of this writing, the patient continues to undergo chemotherapy, and his clinical condition is satisfactory, expect for hypoalbuminemia and bilateral lower limb edema.

## Discussion and conclusion

PIL is a rare condition that is characterized by abnormally dilated intestinal lymphatic channels that leak lymphatic fluid into the gastrointestinal tract; this loss of lymphatic fluid eventually results in hypoproteinemia, hypogammaglobinemia, edema, and lymphocytopenia and other immune abnormalities [1]. PIL was first described in 1961 by Waldmann et al. [2]. The majority of patients with PIL have a benign prognosis, and therefore, the condition is usually diagnosed only late in life. The reported complications of PIL include malignant transformation, cutaneous warts, infections, and gelatinous transformation of the bone marrow [3]. Malignancy, especially lymphoma, as a complication is rarely reported.

In 1972, Waldmann et al. described the relationship between PIL and lymphoma for the first time; among the 50 PIL patients reviewed, 3 had malignant lymphoma [4]. In 1998, Gumà et al. first conducted a retrospective study of 8 patients with small intestinal lymphangiectasis complicated with lymphoma [5]. In 2000, Bouhnik et al. raised the hypothesis that PIL predisposes to lymphoma [6]. Wen et al. retrospectively reviewed 84 PIL patients, and found that 4 (5%) patients developed lymphoma; the mean time of lymphoma detection was 31 years after the PIL diagnosis [7].

We identified a total of 10 studies examining the correlation between PIL and lymphoma. The clinicopathological characteristics of the patients in these studies as well as the patient in the present study (n = 14) are summarized in Table 1 [8–14]. An analysis of the 14 reported cases of PIL-associated lymphoma revealed the following findings: (1) The time from PIL onset to lymphoma detection was 19.14 ± 12.29 years on average (range 3–45 years), and was > 10 years in the majority of patients (79%). (2) In 4 of the 14 patients, the presenting symptom of lymphoma was abdominal pain. This is consistent with the finding of Wen et al. that malignant complications in PIL patients may manifest as an urgent clinical presentation with abdominal pain [7]. The most common sites of PIL-associated lymphoma were the small intestine and stomach, although breast, bone, and retroperitoneal involvement were also observed.

The mechanism underlying the development of lymphoma in patients with PIL may be related to the continuous loss of lymphocytes and immunoglobulins through the intestinal lumen, leading to immune deficiency and weakened immune surveillance [15]. However, patients with PIL have also been reported to have a primary functional B-cell and/or helper T-cell deficiency, and the persistent loss of immunoglobulins and lymphocytes through lymph leakage results in a secondary immune deficiency in these patients [16].

Chemotherapy and radiotherapy dramatically improved the symptoms of both lymphoma and PIL in 4 of the 14 patients. This improvement may be attributable to the inclusion of glucocorticoids in combination chemotherapy regimens, which may have suppressed the inflammatory reactions that would otherwise have led to increased permeability of the intestinal lymphatic vessels [17]. Nevertheless, the PIL symptoms did not completely disappear after the lymphoma treatment in most patients.

In conclusion, PIL is a rare disease with an unclear etiology. A growing body of evidence indicates that the link between PIL and lymphoma is not merely coincidental, and that PIL is a potent predisposing factor for lymphoma after 10 or more years. In some PIL patients, the malignant lesions were confined to the gastrointestinal system, while in others, they were extra-intestinal. The pathophysiological explanation for the association between PIL and lymphoma remains unclear.

**Table 1:** Clinical characteristics of patients with PIL-associated lympho

References	Sex	PIL symptoms	Age at PIL onset (years)	Age at PIL Dx (years)	Lymphoma symptoms	Time to lymphoma* (years)	Lymphoma site	Lymphoma type	Stage	Treatment	Outcome
Strober et al. [15]	–	–	–	–	–	22	Small bowel	–	–	–	–
Waldmann et al. [3]	–	–	–	–	–	3	Breast	–	–	–	–
Waldmann et al. [3]	–	–	–	–	–	25	Stomach	Reticulum cell sarcoma	–	–	–
Ward et al. [8]	M	Peripheral edema	12	29	Acute small bowel obstruction	27	Jejunum	Differentiated lymphocytic lymphoma	I	Surgery, radiotherapy	Died of septicemia
Border et al. [9]	F	Cachexia, diarrhea, vomiting anasarca	24	26	Breast mass	15	Breast	Small non-cleaved cell (B-cell) lymphoma	IV	CVP	Remission of PIL and lymphoma
Massachusetts Hospital*.* [10]	F	Abdominal pain and vomiting	57	57	Dyspnea	13	GI tract, lung, epicardium, mesentery, omentum, pancreas, liver, lymph node, spleen, bone marrow	B-immuno-blastic lymphoma (diffuse histiocytic lymphoma)	IV	VCP	Died of acute broncho-pneumonia
Herait et al. [11]	F	–	3	–	–	15	Retro-peritoneum, mediastinum	Not stated, large cells	IV	AVmCP	Remission of lymphoma, PIL outcome unknown
Shpilberg et al. [12]	F	Diarrhea, vomiting weight loss	6	6	Soft-tissue mass in left thigh	13	Bone	DLBCL	IEB	CHOP, radiotherapy	Remission of PIL and lymphoma
Gumà et al. [5]	F	Tetany, edema	34	34	Abdominal colic, vomiting	20	Jejunum	DLBCL	IE	Surgery, CHOP	Remission of lymphoma, but not PIL
Bouhnik et al. [2]	F	Diarrhea, steatorrhea, abdominal distension	5	11	Abdominal pain	45	Small intestine	B-cell, centroblastic lymphoma	I	Surgery, AVmCP	Remission of lymphoma, but not PIL
Bouhnik et al. [2]	F	Diarrhea, edema	18	58	Abdominal pain	40	Small intestine	B-cell, centroblastic lymphoma	I	PACOB	Remission of lymphoma, but not PIL
Laharie et al. [13]	F	Diarrhea, edema	20	20	Left cervical lymphadeno-pathy	19	Nodal	DLBCL	I	CHOP, radiotherapy	Remission of PIL and lymphoma
Prasad et al. [14]	F	Diarrhea, anasarca	11	17	Abdominal pain and lump	8	Mesenteric	DLBCL	IIIA	R-CHOP, radiotherapy	Remission of PIL and lymphoma
Present case	M	Abdominal distension	34	54	Abdominal pain	23	Colon	DLBCL	IVA	R-CHOP	Remission of lymphoma, but not PIL

*PIL* primary intestinal lymphangiectasia, *Dx* diagnosis, *CVP* cyclophosphamide, vincristine, and prednisone, *VCP* vincristine, chlorambucil, and prednisone, *AVmCP*, adriamycin, teniposide, cyclophosphamide, and prednisone, *CHOP*, cyclophosphamide, adriamycin, vincristine, and prednisone, *PACOB* prednisolone, adriamycin, cyclophosphamide, vincristine, and bleomycin, *R-CHOP* rituximab, cyclophosphamide, doxorubicine, vincristine, and prednisone, *GI* gastrointestinal, *DLBCL* diffuse large B-cell lymphoma

*Time to lymphoma is expressed in terms of years after the onset of PIL

## Data Availability

All data generated or analysed during this study are included in this published article.

## Abbreviations

PIL: Primary intestinal lymphangiectasia
EBER: Epstein-Barr encoding region
CVP: Cyclophosphamide, vincristine, and prednisone
VCP: Vincristine, chlorambucil, and prednisone
AVmCP: Adriamycin, teniposide, cyclophosphamide, and prednisone
CHOP: Cyclophosphamide, adriamycin, vincristine, and prednisone
PACOB: Prednisolone, adriamycin, cyclophosphamide, vincristine, and bleomycin
R-CHOP: Rituximab, cyclophosphamide, doxorubicine, vincristine, and prednisone
GI: Gastrointestinal
DLBC: Diffuse large B-cell lymphoma
